# Rapamycin-mediated mTORC2 inhibition is determined by the relative expression of FK506-binding proteins

**DOI:** 10.1111/acel.12313

**Published:** 2015-02-04

**Authors:** Katherine H Schreiber, Denise Ortiz, Emmeline C Academia, Arieanna C Anies, Chen-Yu Liao, Brian K Kennedy

**Affiliations:** The Buck Institute for Research on Aging8001 Redwood Blvd., Novato, CA, 94945, USA

**Keywords:** aging, FKBP, mTOR, rapamycin

## Abstract

The mechanism by which the drug rapamycin inhibits the mechanistic target of rapamycin (mTOR) is of intense interest because of its likely relevance in cancer biology, aging, and other age-related diseases. While rapamycin acutely and directly inhibits mTORC1, only chronic administration of rapamycin can inhibit mTORC2 in some, but not all, cell lines or tissues. The mechanism leading to cell specificity of mTORC2 inhibition by rapamycin is not understood and is especially important because many of the negative metabolic side effects of rapamycin, reported in mouse studies and human clinical trials, have been attributed recently to mTORC2 inhibition. Here, we identify the expression level of different FK506-binding proteins (FKBPs), primarily FKBP12 and FKBP51, as the key determinants for rapamycin-mediated inhibition of mTORC2. In support, enforced reduction of FKBP12 completely converts a cell line that is sensitive to mTORC2 inhibition to an insensitive cell line, and increased expression can enhance mTORC2 inhibition. Further reduction of FKBP12 in cell lines with already low FKBP12 levels completely blocks mTORC1 inhibition by rapamycin, indicating that relative FKBP12 levels are critical for both mTORC1 and mTORC2 inhibition, but at different levels. In contrast, reduction of FKBP51 renders cells more sensitive to mTORC2 inhibition. Our findings reveal that the expression of FKBP12 and FKBP51 is the rate limiting factor that determines the responsiveness of a cell line or tissue to rapamycin. These findings have implications for treating specific diseases, including neurodegeneration and cancer, as well as targeting aging in general.

## Introduction

Mechanistic target of rapamycin (mTOR) is a nutrient responsive kinase that integrates signals from growth factors, amino acids, energy, and stress to regulate cell growth and proliferation (Zoncu *et al*., 2011; Dibble & Manning, 2013). mTOR is the catalytic subunit of two distinct complexes, mTORC1 and mTORC2; the former regulates protein and lipid synthesis, as well as cellular stress response pathways, and the latter modulates cell survival. mTORC1 primarily controls protein synthesis through the direct phosphorylation of both S6 Kinase 1 (S6K1) and 4E (eIF4E)-binding protein 1 (4E-BP1). In addition to regulating protein synthesis, mTORC1 activates lipogenesis and negatively regulates autophagy. mTORC2 signaling is much less understood, but is known to be activated by growth factors, including insulin through PI3K. mTORC2 activates several AGC subfamily kinases, including Akt, SGK1, and PKC-α which regulate cellular processes, including cell survival, proliferation, and cytoskeletal organization (Laplante & Sabatini, 2012).

Rapamycin, a drug currently approved for multiple disease indications, extends lifespan in yeast (Kaeberlein *et al*., 2005b; Powers *et al*., 2006), flies (Kapahi & Zid, 2004; Bjedov *et al*., 2010), worms (Vellai *et al*., 2003), and mice (Chen *et al*., 2009; Harrison *et al*., 2009). Currently, the effectors downstream of mTOR that regulate the longevity actions of this drug are unknown, but are thought to involve mTORC1 substrates as S6K1^−/−^ leads to lifespan extension in a range of animal models including mice (Fabrizio *et al*., 2001; Kapahi *et al*., 2004; Kaeberlein *et al*., 2005a; Hansen *et al*., 2007; Pan *et al*., 2007; Selman *et al*., 2009), and 4EBP mutant flies are long-lived (Kapahi *et al*., 2010). While rapamycin acutely and directly inhibits mTORC1 (Heitman *et al*., 1991; Kim *et al*., 2002), chronic administration *in vivo* or in cell culture can inhibit mTORC2 through a mechanism proposed to involve inhibition of complex assembly (Sarbassov *et al*., 2006; Lamming *et al*., 2012). In addition to some of the beneficial effects of rapamycin on longevity and in disease models (Stanfel *et al*., 2009; McCormick *et al*., 2011; Zoncu *et al*., 2011), chronic administration of rapamycin is associated with detrimental effects on metabolism, including hyperglycemia, hyperlipidemia, and insulin resistance in mice (Fraenkel *et al*., 2008; Houde *et al*., 2010), and a similar metabolic profile has been shown in humans treated with rapamycin (Stallone *et al*., 2009). Recently, mTORC2 inhibition, at least in part in the liver, was found to mediate some of these effects in mice (Lamming *et al*., 2012). To effectively design new therapies with the beneficial effects of rapamycin and fewer adverse side effects, it will be critical to understand the different mechanisms leading to the inhibition of these two complexes.

It is well established that rapamycin directly inhibits mTORC1 through a noncompetitive mechanism by establishing a three-way interaction with mTOR and FKBP12 (Liang *et al*., 1999). While rapamycin binds mTORC1 directly, it does not bind the mTORC2 complex. Instead, it is proposed that rapamycin inhibits the assembly of the mTORC2 complex in a manner requiring FKBP12. Until recently, FKBP12 was thought to be the primary FKBP which binds rapamycin, but other FKBPs, including FKBP51 and FKBP52, have been shown to form complexes with mTOR in the presence of rapamycin (Marz *et al*., 2013). However, the role of the different FKBP–rapamycin complexes on mTORC1 and mTORC2 activity has not been fully elucidated.

While mTORC1 inhibition by rapamycin is universal, only 15 of 39 cell lines tested were responsive to mTORC2 inhibition by rapamycin (Sarbassov *et al*., 2006). The mechanism leading to these differences is not understood and is of great interest because of the implications of rapamycin in the treatment of many disease including cancer (Wander *et al*., 2011), Alzheimer's disease (King *et al*., 2008), heart disease (Flynn *et al*., 2013), and the aging process in general. In the context of aging, specific inhibition of mTORC1 may reduce side effects of rapamycin, possibly allowing for higher dosing and/or prolonged treatment of the drug, which may maximize the effects on aging and enable translation to human studies. Understanding what causes one cell line or tissue to be responsive to mTORC2 inhibition by rapamycin will therefore have clinical relevance, defining the disease indications that might be responsive to rapamycin (and derivatives). Here, we identify the expression levels of FKBP12 and FKBP51 as key determinants in defining whether a cell line or tissue is responsive to mTORC2 inhibition by rapamycin.

## Results

**Figure 1:**
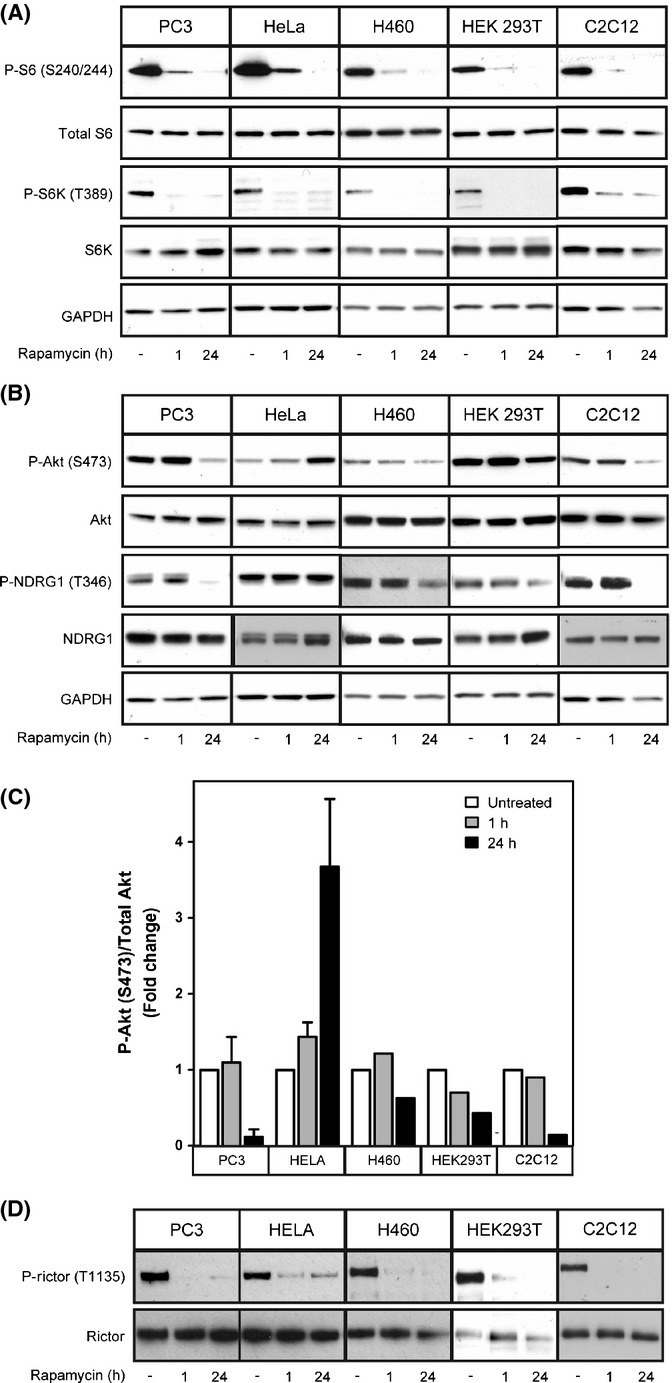
Rapamycin differentially inhibits mTORC2 in various cell lines. PC3, HeLa, HEK 293T, H460, or C2C12 cells were treated with 100 nm rapamycin for 1 or 24 h. The phosphorylation of specific mTORC1 (P-S6K T389, P-S6 S240/244) (A) and mTORC2 (P-Akt S473, P-NDRG1 T346) substrates (B) was examined by Western blot analysis. The responsiveness of each cell line to mTORC2 inhibition by rapamycin is quantified (C). The phosphorylation of the mTORC1 substrate, Rictor, is inhibited by rapamycin in PC3, HeLa, HEK 293T, H460, and C2C12 cells to a similar extent (D).

Many cell lines, such as PC3 cells, are responsive to mTORC2 inhibition by rapamycin, while others, including HeLa cells, are nonresponsive (Sarbassov *et al*., 2006). To confirm these differences, we treated five different cell lines with rapamycin for 1 (acute) or 24 hours (chronic). While rapamycin inhibits mTORC1 to approximately the same extent in all cell lines tested at both time points (Figs1A and S1), a 24-h rapamycin treatment inhibits mTORC2 signaling tenfold in PC3 cells and C2C12 cells, fourfold in HEK 293T cells and H460 cells, and in contrast causes a fourfold increase in the phosphorylation of the mTORC2 substrate, P-Akt (S473) in HeLa cells (Fig.1B,C). Activation of mTORC2 signaling likely results from loss of S6 kinase-mediated feedback inhibition of the insulin/IGF pathway and/or Rictor in cells where rapamycin acts in an mTORC1-specific fashion. A similar pattern is seen for the inhibition of an indirect substrate of mTORC2, P-NDRG1 (T346), which is often used as a measure of mTORC2 activity. Whereas PC3 and C2C12 cells show the most robust inhibition of P-NDRG1, H460 and HEK 293T cells show modest inhibition of P-NDRG1, and HeLa cells show no inhibition of P-NDRG1 (Fig.1B).

A simple explanation for the difference between cells lines with respect to mTORC2 inhibition by rapamycin could be impairment in mTOR's intrinsic negative feedback loop, described above. mTORC1-activated S6 kinase negatively regulates mTORC2 by two mechanisms: by phosphorylating IRS leading to decreased insulin signaling and subsequent Akt phosphorylation and through the phosphorylation of Rictor, leading to the subsequent inactivation of Akt (Harrington *et al*., 2004; Dibble *et al*., 2009; Julien *et al*., 2010). To assess whether a faulty negative feedback loop could explain differences among cell lines, we examined the phosphorylation of Rictor (T1135) in all five cell lines. Inhibition of the phosphorylation of Rictor following a 24-h rapamycin treatment was seen in all cases, regardless of the response to mTORC2 inhibition, indicating that disruption of this negative feedback loop cannot explain the differences in cell lines with respect to mTORC2 inhibition (Figs1D and S1).

**Figure 2:**
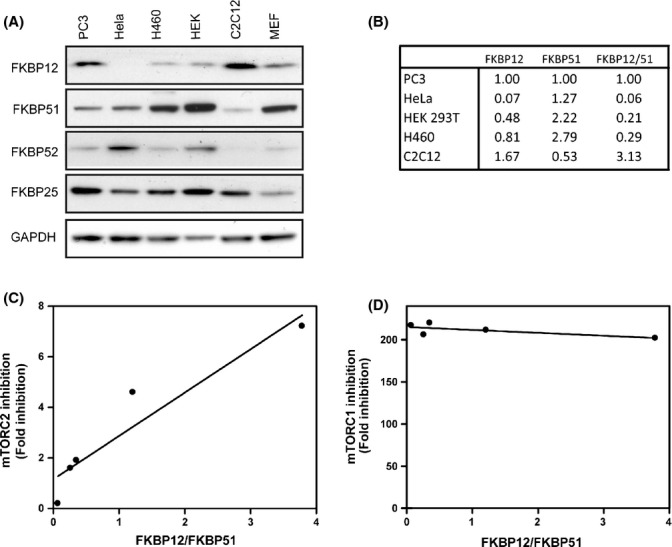
FKBP12/FKBP51 ratio determines cell's responsiveness to mTORC2 inhibition by rapamycin. Expression of FKBP12, 25, 51, and 52 was examined in PC3, HeLa, H460, HEK 293T, C2C12, and MEFs (A). The ratio of FKBP12 to FKBP51 was quantified from each cell line (B). The correlation between the responsiveness of each cell line to mTORC2 inhibition and FKBP12/51 ratio is plotted (C). The correlation between the responsiveness of each cell line to mTORC1 inhibition and FKBP12/51 ratio is plotted (D).

Why then is rapamycin-mediated inhibition of mTORC2 signaling restricted to some cell lines? It has been established that the expression of some of the core components of the mTOR complexes (e.g., mTOR, Raptor, and Rictor) do not change between cell lines and the differences in responsiveness to rapamycin can also not be attributed to PTEN expression (Sarbassov *et al*., 2006). FKBPs are another integral component of mTOR inhibition by rapamycin, and recently, FKBPs besides FBKP12 were identified as binding mTOR (Marz *et al*., 2013). We reasoned that specific FKBPs may have differential binding affinities for the different mTOR complexes and that their expression may affect the cellular responsiveness to rapamycin. To test whether FKBP expression changes among cell lines that respond differently to mTORC2 inhibition by rapamycin, we examined the expression of four FKBPs known to interact with rapamycin, FKBP12, 25, 51, and 52, in each of the cell lines previously described. FKBP12 expression is highest in cell lines that are most responsive to mTORC2 inhibition by rapamycin, PC3, and C2C12 cells (Fig.2A,B) while, in contrast, FKBP51 and FKBP52 are expressed to a lesser extent. Cell lines that have intermediate responsiveness to mTORC2 inhibition, HEK 293T and H460 cells, display intermediate levels of FKBP12 and FKBP51, while cells that show no inhibition of mTORC2 (HeLa cells) show very low levels of FKBP12 and relatively high levels of FKBP51 (Fig.2C). Quantification of each of the FKBPs reveals that the higher the FKBP12/FKBP51 ratio, the more responsive the cell line is to mTORC2 inhibition (Fig.2C) (*R*^2^ = 0.8962), while inhibition of mTORC1 is not affected by this ratio (Fig.2D) (*R*^2^ = 0.5101).

**Figure 3:**
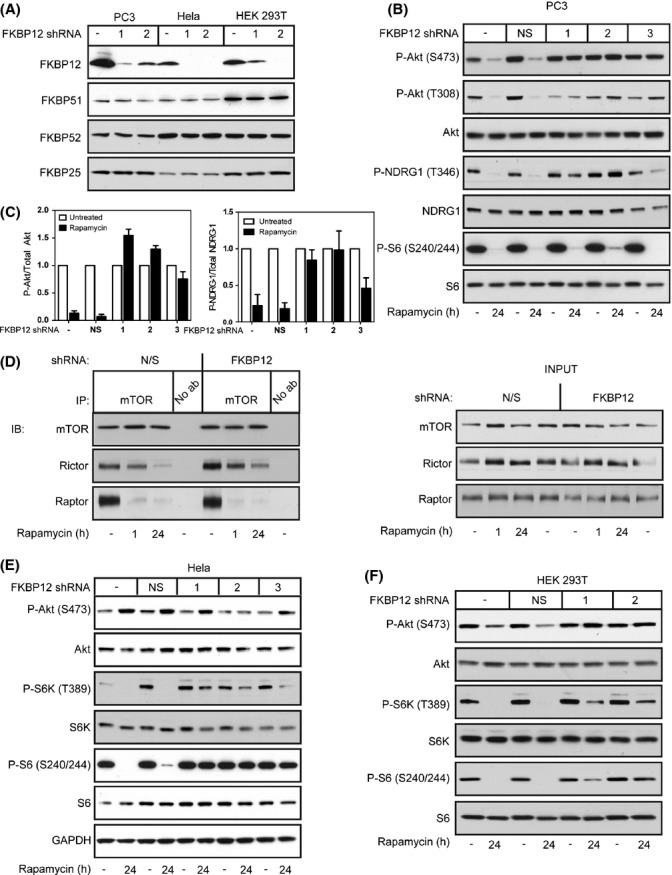
Knockdown of FKBP12 impairs mTORC2 inhibition by rapamycin. shRNA constructs directed toward FBKP12 were transfected into PC3, HeLa, or HEK 293T cells (A). PC3 cells expressing FKBP12 shRNA 1, 2, or 3 were treated with rapamycin for 24 h, and the effects of reducing FKBP12 levels on mTORC1 signaling (P-S6 S240/244) and mTORC2 signaling (P-Akt S473 and P-NDRG1 T346) were examined by Western blot analysis (B) and quantified (C). Immunoprecipitations for mTOR were performed in PC3 cells in the presence or absence of FKBP12 shRNA, and Western blot analysis was performed for mTOR, Rictor, and Raptor (D). HeLa (E) or HEK 293T cells (F) expressing FKBP12 shRNA 1, 2, or 3 were treated with rapamycin for 24 h, and the effects of reducing FKBP12 levels on mTORC1 signaling (P-S6K T389 and P-S6 S240/244) and mTORC2 signaling (P-Akt S473) were examined by Western blot analysis. Results are the average of three independent experiments ± SEM.

As FKBP12 expression correlates with a higher responsiveness to mTORC2 inhibition, we examined whether shRNA-enforced reduction in FKBP12 would convert an otherwise rapamycin-mediated mTORC2 sensitive cell line to a resistant one. We transfected PC3 cells with five different shRNA constructs targeting FKBP12. Three of these constructs gave robust knockdown of FKBP12 expression to levels comparable to that described for HeLa cells (Fig.3A). The expression of FKBP51, 52, and 25 was unaffected by FKBP12 shRNAs. PC3 cells expressing the shRNA constructs were then treated with rapamycin for 24 h, and the effects on mTORC1 and mTORC2 inhibition were determined. Strikingly, knockdown of FKBP12 by all three shRNAs completely blocked the inhibition of mTORC2 by rapamycin, as measured by the phosphorylation of the mTORC2 targets Akt (S473 and T308) and NDRG1 (T346) in PC3 cells (Fig.3B,C). Dissociation of Rictor from mTOR caused by a 24-h rapamycin treatment is also directly blocked by the reduction in FKBP12 as determined by co-immunoprecipitation (Fig.3D), confirming that reduction of FBKP12 expression impairs the inhibition of mTORC2 by rapamycin. In contrast, knockdown of FKBP12 alone had no effect on mTORC1 activity in PC3 cells in the presence of rapamycin, as measured by the phosphorylation of S6 (S240/244) (Fig. 3B) or the dissociation of Raptor (Fig. 3D).

In accord with the findings from Marz and colleagues (Marz *et al*., 2013), other FKBPs may compensate for the reduction in FKBP12 expression with respect to rapamycin-mediated mTORC1 inhibition. Alternatively, the level of knockdown of FKBP12 in PC3 cells may not be sufficient to impact mTORC1 signaling, as indicated by the fact that the levels are only lowered to the basal levels of HeLa cells, which are still responsive to mTORC1 inhibition by rapamycin. To distinguish between these two possibilities, we tested whether lowering FKBP12 levels to an even greater extent in cell lines which already have low FKBP12 levels, HeLa and HEK 293T (Fig.3A), would affect mTORC1 inhibition by rapamycin. Reduction of FKBP12 in HeLa cells completely prevents mTORC1 inhibition by rapamycin, as measured by P-S6K (T389) and P-S6 (S240/244) (Fig.3E). Similarly, reduction of FKBP12 levels in HEK 293T cells, which display slightly higher levels of basal FKBP12 compared to HeLa cells (Fig.3A), completely blocks the inhibition of both mTORC1 and mTORC2 by rapamycin (Fig.3F). These results imply that there is a threshold of FKBP12 expression that is required for mTORC1 inhibition by rapamycin, where a higher expression level of FKBP12 is required for mTORC2 inhibition.

**Figure 4:**
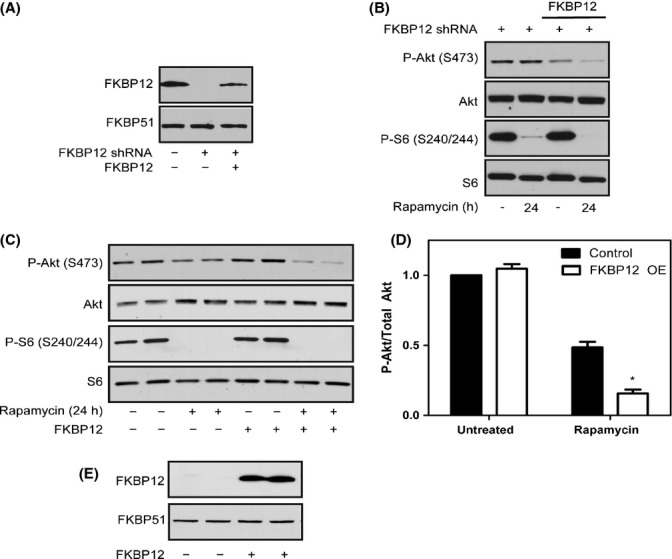
Overexpressing FKBP12 sensitizes HEK 293T cells to mTORC2 inhibition by rapamycin. An FKBP12 lentiviral construct was introduced into FKBP12-shRNA expressing PC3 cells (A). PC3 cells expressing the shRNA construct in the presence or absence of the FKBP12 construct were treated with rapamycin for 24 h, and mTORC1 activity (P-S6 S240/244) and mTORC2 activity (P-Akt S473) were examined by Western blot analysis (B). The FKBP12 lentiviral construct was transfected into HEK 293T cells, and cells were treated with or without rapamycin for 24 h. Western blot analysis was performed to examine mTORC1 activity (P-S6 S240/244) and mTORC2 activity (P-Akt S473) (C) and quantified (D). FKBP12 expression was analyzed in HEK 293T cells +/− the FKBP12 construct (E). Results are the average of four independent experiments ± SEM. Statistical significance is indicated (*P < 0.001 compared to untreated control).

To verify that FKBP12 expression is one of the main indicators for mTORC2 responsiveness, we examined the effects of overexpressing FKBP12 in nonresponsive cell lines. To confirm that our exogenously expressed FKBP12 is functional, we reintroduced FKBP12 into FKBP12 shRNA expressing PC3 cells, which are completely unresponsive to mTORC2 inhibition (Figs3B and 4B). FKBP12 expression restored the protein to nearly basal levels (Fig.4A) and resensitized FKBP12 shRNA expressing PC3 cells to mTORC2 inhibition by rapamycin (Fig.4B), confirming that our overexpressed FKBP12 is functional. To test whether overexpressing FKBP12 would convert an mTORC2 insensitive cell line to a sensitive one, we overexpressed FKBP12 in HEK 293T cells (Fig.4E). Overexpressing FKBP12 sensitizes HEK 293T cells to mTORC2 inhibition by rapamycin as measured by the downstream target of mTORC2, P-Akt (S473) (Fig.4C,D). Taken together, these results indicate that FKBP12 expression is critical in determining the responsiveness of cells to mTORC2 inhibition by rapamycin.

**Figure 5:**
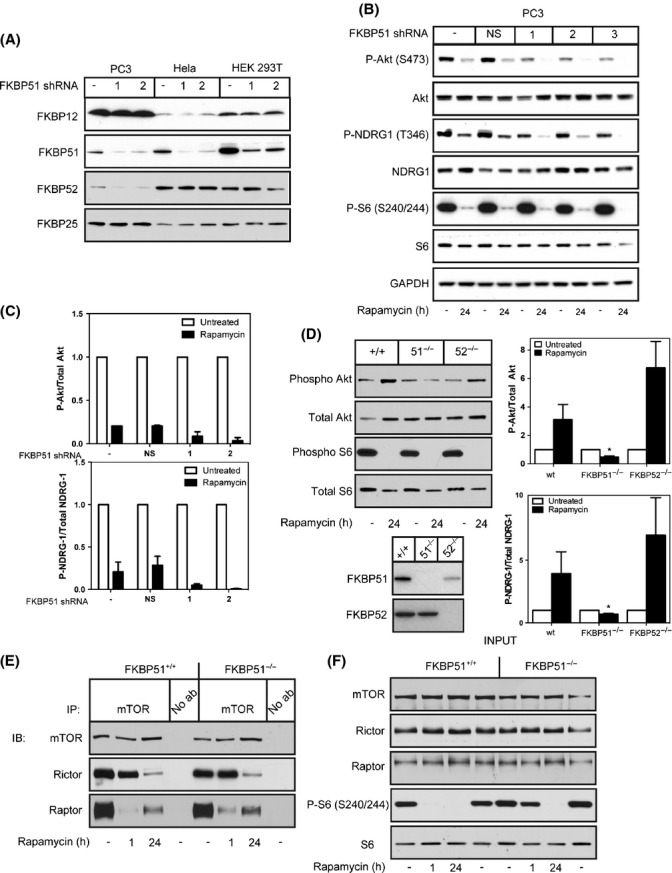
Knockdown of FKBP51 renders cell lines more responsive to mTORC2 inhibition. shRNA constructs directed toward FBKP51 were transfected into PC3, HeLa, or HEK 293T cells (A). PC3 cells expressing shRNA 1, 2, or 3 were treated with rapamycin for 24 h, and the effects of knocking down FKBP51 on mTORC1 signaling (P-S6 S240/244) and mTORC2 signaling (P-Akt S473 and P-NDRG1 T346) were examined by Western blot analysis (B) and quantified (C). Wild-type,*FKBP51*^*−/−*^, or *FKBP52*^*−/−*^ MEFs were treated with rapamycin for 24 h, and their responsiveness to mTORC1 (P-S6 S240/244) and mTORC2 (P-Akt S473) was evaluated by Western blot analysis and quantified (D). Results are the average of five independent experiments ± SEM. Immunoprecipitations were performed using an anti-mTOR antibody on wild-type or *FKBP51*^−/−^ MEFs. Immunoprecipitated samples were then immunoblotted for mTOR, Rictor, or Raptor (E). Input samples were analyzed for mTOR, Raptor, Rictor, P-S6 (S240/244), or S6 (F). Results are the average of 3-5 independent experiments ± SEM. Statistical significance is indicated (*P < 0.001 compared to untreated control).

Because FKBP51 and FKBP52 can also form complexes with rapamycin and mTOR (Marz *et al*., 2013), and low FKBP51 expression correlates with mTORC2 inhibition by rapamycin, we rationalized that these proteins may compete with FKBP12 for rapamycin and, therefore, the ratio of FKBP12 to FKBP51 or FKBP52 could be critical in determining a cell's responsiveness to mTORC2 inhibition by rapamycin. To test whether FKBP51 contributes to a cell's responsiveness to mTORC2 inhibition by rapamycin, we reduced levels of FKBP51 with five different shRNA constructs in PC3 cells (Fig.5A). PC3 cells in this context displayed enhanced inhibition of the mTORC2 substrates P-Akt (S473) and P-NDRG1 (T346) after 24-h rapamycin treatment (Fig.5B,C). Reduction of FKBP51 in HeLa cells or HEK 293T cells has no effect on mTORC2 inhibition by rapamycin, potentially because the reduction of FKBP51 is not great enough to shift the ratio of FKBP12/FKBP51 to that of a responsive cell line (Fig. S2).

In addition, *FKBP51*^−*/*−^ MEFs are more sensitive to mTORC2 inhibition by rapamycin as measured by the phosphorylation of downstream targets of mTORC2, Akt (S473) and NDRG1 (T346), compared to wild-type MEFs, whereas *FKBP52*^−*/*−^ MEFs behaved similarly to wild-type controls (Fig.5D). However, co-immunoprecipitation experiments reveal that Rictor dissociation occurs to the same extent in the presence or absence of FKBP51, indicating that FKBP51 does not directly affect mTORC2 dissociation (Fig.5E). Instead, we found that Raptor association with mTOR was slightly increased in the *FKBP51*^−*/*−^ cells with rapamycin treatment compared to *FKBP51*^*+/+*^ controls and this was confirmed by a partial blockage of mTORC1 inhibition at 1-h rapamycin in *FKBP51*^−*/*−^ cells as measured by P-S6 (S240/244) (Fig.5E,F). Interestingly, there was no observed effect on mTORC1 inhibition by rapamycin at 24 h. These results support a role for FKBP51 in the regulation of mTORC1 directly, consistent with the study by Marz *et al*. (Marz *et al*., 2013), and now, we have identified that expression levels of FKBP51 impact the inhibition of mTORC2 by rapamycin as well.

**Figure 6:**
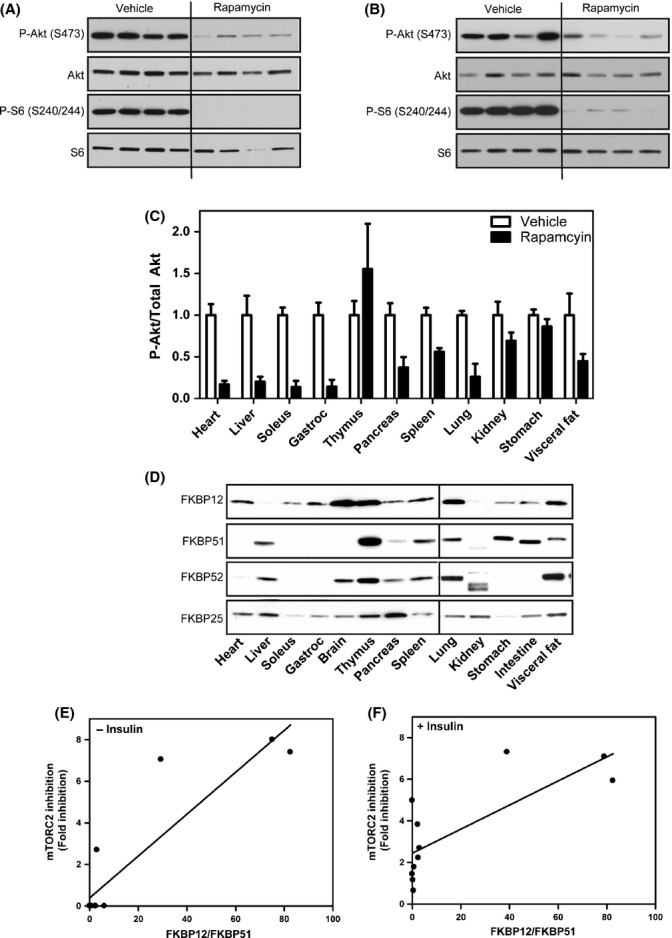
FKBP expression in mouse tissues correlates with responsiveness to mTORC2 inhibition by rapamycin. Mice were IP injected with rapamycin (8 mg kg^−1^) every other day for 3 weeks. Mice were fasted overnight and injected for 15 min with insulin, and the responsiveness of each tissue to mTORC1 (P-S6 S240/244) or mTORC2 (P-Akt S473) inhibition by rapamycin was examined. Heart (A) and liver (B) are responsive to mTORC2 inhibition. Full characterization of other tissues is shown in Fig. S3 and quantified (C). Expression of FKBPs in mouse tissues was evaluated by Western blot analysis (D). Correlation between FKBP12/FKBP51 and mTORC2 inhibition in each tissue was evaluated under different tissue harvesting conditions: 6-h fast (E) or an overnight fast followed by 15-min insulin injection (F). Results are the average of 4 individual mice per cohort ± SEM.

It is well described that rapamycin leads to the inhibition of mTORC1 *in vivo*, but the effects of rapamycin on mTORC2 in individual tissues have not been clearly established and is critical to understand the effects of rapamycin *in vivo*. To address this issue, we IP injected 10-week-old mice with 8 mg kg^−1^ rapamycin every other day for 3 weeks. Mice were fasted overnight and treated with insulin for 15 min prior to tissue harvest, as described by Lamming *et al*. (2012) to optimize for mTORC2 inhibition. As expected, inhibition of the phosphorylation of the mTORC1 substrate, S6 (S240/244), was seen in every tissue tested, but mTORC2 inhibition, measured by the phosphorylation of Akt (S473), was only seen to differing degrees in a subset of tissues including heart, soleus muscle, gastrocnemius muscle, pancreas, liver, lung, visceral fat, and spleen (Figs6A,B,C and S3). mTORC2 inhibition was heightened by these harvesting conditions, because under harvesting conditions where mice were starved for only 6 h without insulin stimulation, only heart, soleus, gastrocnemius, and pancreas are responsive to mTORC2 inhibition (Fig. S4). Tissues that are completely unresponsive to mTORC2 inhibition under either experimental condition include thymus, kidney, and stomach (Figs6C, S3 and S4).

To test whether the differential response of the various tissues to mTORC2 inhibition by rapamycin is due to altered levels of FKBPs, we examined their expression in multiple tissues. FKBP12 and FKBP51 levels have been reported in human tissues (Baughman *et al*., 1997), but data in mouse tissues are largely unavailable. Similar to the cell lines, we see a range of FKBP expression among tissues (Fig.6D). As with the cell lines, a higher FKBP12/FKBP51 ratio in tissues correlates with responsiveness to mTORC2 inhibition by rapamycin under either harvesting condition: a 6-h fast (Fig.6E) (*R*^2^ = 0.8196) or an overnight fast with 15-min insulin stimulation (Fig.6F) (*R*^2^ = 0.6048), further supporting the hypothesis that FKBP12 is required for inhibition of mTORC2 *in vivo*.

## Discussion

In this study, we have discovered that the relative expression of FKBP12 and FKBP51 determines a cell or tissue's ability to respond to mTORC2 inhibition by rapamycin. When FKBP12 expression is high compared to other FKBPs, rapamycin through FKBP12 binding will lead to the inhibition of mTORC2 complex formation. When other FKBPs compete with FKBP12 for rapamycin, the effective inhibition of mTORC2 decreases. These findings provide a new mechanistic understanding of how different FKBPs regulate the cellular response to rapamycin and points to the level of FKBPs as the rate limiting factor determining cellular responsiveness to rapamycin.

Reduction of FKBP12 in PC3 cells completely prevents the inhibition of mTORC2 by rapamycin, while having no effect on mTORC1 inhibition, suggesting that FKBP12 alone is required for mTORC2 inhibition, but not necessary, at least at high levels, for mTORC1 inhibition. However, when reducing FKBP12 expression in HeLa cells, which already have very low endogenous levels of FKBP12, cells are no longer responsive to mTORC1, indicating that there is a threshold of FKBP12 expression levels that is required in order for cells to respond to mTORC1 inhibition by rapamycin; high FKBP12 levels support mTORC2 inhibition, whereas lower levels are sufficient for mTORC1 inhibition. Furthermore, we found that overexpressing FKBP12 sensitizes HEK 293T cells to mTORC2 inhibition by rapamycin. While we have attempted to examine the effects of overexpressing FKBP12 in HeLa cells as well, we have not had success in expressing FKBP12 to the same extent in this cell line (data not shown). As we have demonstrated that the level of FKBP12 is critical to cellular responsiveness to mTORC2 inhibition by rapamycin, we postulate that it will be imperative to obtain similar expression levels of functional FBKP12 to that in PC3 cells to sensitive HeLa cells to mTORC2 inhibition.

**Figure 7:**
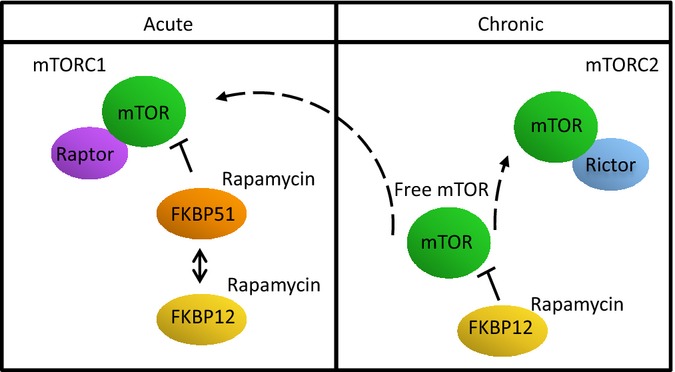
FKBP51 competes with FKBP12 for acute inhibition of mTORC1, while FKBP12 is required for prolonged inhibition of mTORC1 and mTORC2.

In addition to FKBP12 levels, we show that FKBP51 levels also play a role in the responsiveness to mTORC2 inhibition. *FKBP51*^*−/−*^ MEFs are more sensitive to mTORC2 inhibition, but Rictor dissociation is not directly affected, indicating that FKBP51 indirectly affects mTORC2 complex formation. Instead *FKBP51*^*−/−*^ MEFs have impaired inhibition of mTORC1, which may in turn be affecting mTORC2 activity either through feedback regulation by the mTORC1 pathway on mTORC2 or simply through competing with FKBP12 for rapamycin. These results point to a model where FBKP12 and FKBP51 are required for the direct inhibition of mTORC1, while FKBP12 is the only FKBP which can bind free mTOR leading to inhibition of both mTORC1 and mTORC2 after prolonged exposure to the drug (Fig.7).

In addition to detailing the molecular interplay between FKBP12 and FKBP51 *in vitro*, we characterize rapamycin-mediated inhibition of mTORC1 and mTORC2 *in vivo*. Using 2 different experimental procedures, we confirm that mTORC2 inhibition occurs in some, but not all tissues, similar to the response seen in cell lines (Sarbassov *et al*., 2006). Heart, skeletal muscle, and pancreas are the most responsive tissues to mTORC2 inhibition, while thymus, kidney, and stomach were the least responsive to mTORC2 inhibition. Knowing which tissues are responsive to mTORC1 and mTORC2 inhibition by rapamycin is critical to understand some of the whole body side effects associated with rapamycin treatment. For example, the negative effects of rapamycin on glucose homeostasis have been attributed to the inhibition of mTORC2 in the liver (Lamming *et al*., 2012), but the contributing effects of mTORC2 inhibition in other key tissues, including pancreas, are unknown. Here, we reproducibly characterize the degree of mTORC2 inhibition by rapamycin across tissues.

Identification of FKBP levels as a key determinant for cellular responsiveness to mTORC2 inhibition is potentially critical in treating diseases with rapamycin, particularly cancer. mTORC1 and mTORC2 have both been implicated in different cancers, yet rapamycin is not effective at treating them all (Wander *et al*., 2011; Laplante & Sabatini, 2012). Our data suggest that differences in responsiveness to rapamycin could be due to expression levels of the different FKBP proteins, specifically FKBP12 and FKBP51. This identifies the FKBP12:51 ratio as a potential biomarker that predicts which tumors/cancers will be responsive to rapamycin. PTEN was identified as a potential biomarker (Neshat *et al*., 2001), because PTEN null cells were more responsive to mTORC1 inhibition. However, PTEN loss does not correlate with responsiveness to mTORC2 inhibition (Sarbassov *et al*., 2006), and in the clinical setting, PTEN has proven an inadequate marker.

In addition to cancer, rapamycin has been implicated in the prevention of a range of chronic disease, including Alzheimer's, cardiovascular syndromes, and many others, as well as an agent that can delay aging in mice (Harrison *et al*., 2009; Stanfel *et al*., 2009; Flynn *et al*., 2013). Understanding the role of the two complexes in each of these diseases will be critical in determining the efficacy of rapamycin toward a particular disease. Of importance is the fact that many of the negative side effects of rapamycin on glucose and lipid homeostasis have been attributed to mTORC2 inhibition (Lamming *et al*., 2012); therefore, new mTORC1 selective inhibitors could be identified that would be more beneficial in some clinical settings than others.

In some tissues/diseases, such as cancer, it will likely be beneficial to inhibit both mTORC1 and mTORC2 where others, such as the aging process in general, might benefit from mTORC1-selective inhibition to avoid the negative metabolic side effects associated with prolonged treatment of the drug (Stallone *et al*., 2009; Houde *et al*., 2010). Current literature supports that the inhibition of mTORC1 contributes to the longevity effects of rapamycin and the inhibition of mTORC2, specifically in the liver, causes the negative effects of rapamycin on glucose homeostasis. However, more experiments are required to understand how the inhibition of these 2 complexes on a tissue-by-tissue basis affects the longevity of the organism as a whole. In this study, we provide an initial examination of the responsiveness of each tissue to mTORC2 inhibition and attribute the levels of FKBP12 and FKBP51 as determining factors for this responsiveness. It is clear that a robust understanding of the effects of rapamycin in each tissue and the mechanism underlying that response is essential for use of this drug and derivatives to prolong human aging.

## Experimental procedures

### Cell culture

PC3 cells were maintained in F12K media supplemented with 10% FBS, 1% penicillin/streptomycin, and 2 mm l-glutamine. HeLa, HEK 293T, C2C12, and MEF cells were maintained in DMEM supplemented with 10% FBS, 1% penicillin/streptomycin, and 2 mm l-glutamine. H460 cells were maintained in RPMI media supplemented with 10% FBS, 1% penicillin/streptomycin, and 2 mm l-glutamine. *FKBP51*^*−/−*^, *FKBP52*^*−/−*^, and their littermate controls were a kind gift of Weinian Shou (Indiana University). All cell lines were cultured at 37 °C under an atmosphere of 95% air and 5% CO_2_. Following treatments, cells were harvested in RIPA buffer [300 mm NaCl, 1.0% NP-40, 0.5% sodium deoxycholate, 0.1% SDS, 50 mm Tris (pH 8.0), protease inhibitor cocktail (Roche, Mannheim, Germany), phosphatase inhibitor 2, 3 (Sigma, St. Louis, MO)] and protein concentrations were determined using the DC protein assay (Bio-Rad, Hercules, CA).

### Lentiviral transduction

Lentiviral pGIPZ shRNA constructs were purchased from Thermo Scientific (Waltham, MA). Briefly, HEK 293T cells were transfected with pGIPZ constructs bearing shRNA against FKBP12 or FKBP51 or a nonspecific control using calcium phosphate transfection as previously described (O'Leary *et al*., 2013). After 48 h, viral supernatant was harvested and used to transduce PC3, HeLa, or HEK 293T cells. Cells were selected for 5 days in puromycin (2 μg mL^−1^) before experimentation.

### Western blot analysis

Equal amounts of protein were resolved by SDS-PAGE and transferred to nitrocellulose membrane using the Invitrogen Nu-Page system (Carlsbad, CA). Western blot analysis was performed using the following protein-/phospho-protein-specific antibodies: anti-p70 S6 kinase (#2708), anti-phospho-p70 S6 kinase (T389) (#9234), anti-rpS6 (#2217), anti-phospho-rpS6(S240/244) (#5364), anti-phospho-NDRG-1 (T346) (#5482), anti-NDRG-1 (#9485), anti-phospho-Akt (S473) (#4060), anti-Akt (#4691), anti-FKBP51 (#12210), anti-FKBP52 (#11826), anti-mTOR (#2972), anti-Rictor (#9476), anti-phospho-Rictor (T1135) (#3806), and anti-Raptor (#2280) were from Cell Signaling Technologies (Danvers, MA). Anti-FKBP25 (ab16654) and anti-FKBP12 (ab2918) were from Abcam. Anti-GAPDH (AM4300) was from Ambion Austin, TX.

### Immunoprecipitation

Cells were lysed in 0.3% CHAPS lysis buffer (40 mm Hepes, pH 7.5, 120 mm NaCl, 1 mm EDTA, 0.3% CHAPS, 10 mm pyrophosphate, 10 mm β-glycerophosphate, 50 mm NaF, 0.5 mm orthovanadate, protease inhibitor tablet) as described (Sarbassov *et al*., 2006). 1.2 mg of protein was incubated with or without an anti-mTOR antibody (Cell Signaling #2972) overnight at 4 °C on a nutator. The next day, lysate–antibody mixtures were conjugated to magnetic protein-G beads (Pierce, Rockford, IL, Cat # 88848) for 2 h at 4 °C on a nutator. Beads were washed three times with 0.3% CHAPS lysis buffer. Thirty milcroliters of 2× SDS buffer was added to the beads, and the samples were boiled for 5 min. Thirty micrograms of each sample was used as an input control.

### Animals/tissue preparation

Ten-week-old C57BL/6J mice were given intraperitoneal injections of 8 mg kg^−1^ rapamycin (LC laboratories, Woburn, MA) or vehicle every other day for 10 days (4–5 mice/group). Mice were fasted overnight (16 h) following the last rapamycin injection and injected with insulin for 15 min just before tissue harvesting. Tissues were immediately frozen in liquid nitrogen. Alternatively, tissues were dissected from mice that were fasted for 6 h and immediately frozen in liquid nitrogen. The tissues were homogenized using the Omni TH homogenizer (Omni International, Kennesaw, GA) on ice in RIPA buffer [300 mm NaCl, 1.0% NP-40, 0.5% sodium deoxycholate, 0.1% SDS, 50 mm Tris (pH 8.0), protease inhibitor cocktail (Roche), phosphatase inhibitor 2, 3 (Sigma)] and then centrifuged at 21,130 × g for 15 min at 4 °C. The supernatants were collected, and protein concentration was determined using the DC protein assay (Bio-Rad).

## Supporting Information

**Fig. S1** Rapamycin differentially inhibits mTORC2 in various cell lines. **Fig. S2** Knockdown of FKBP51 has no effect on mTORC2 inhibition by rapamycin in HeLa or HEK 293T cells. **Fig. S3** Inhibition of mTORC2 by rapamycin *in vivo* following an overnight fast and stimulation with insulin. **Fig. S4** Inhibition of mTORC2 by rapamycin *in vivo* following a 6 h fast.
