# Supplementary material for: Rapamycin-mediated mTORC2 inhibition is determined by the relative expression of FK506-binding proteins
Source: Aging Cell. 2015 Feb 4;14(2):265–73. doi: 10.1111/acel.12313 (PMC4364838; doi:10.1111/acel.12313)
Supplement: Supplementary file 1 [file acel0014-0265-sd1.pdf]

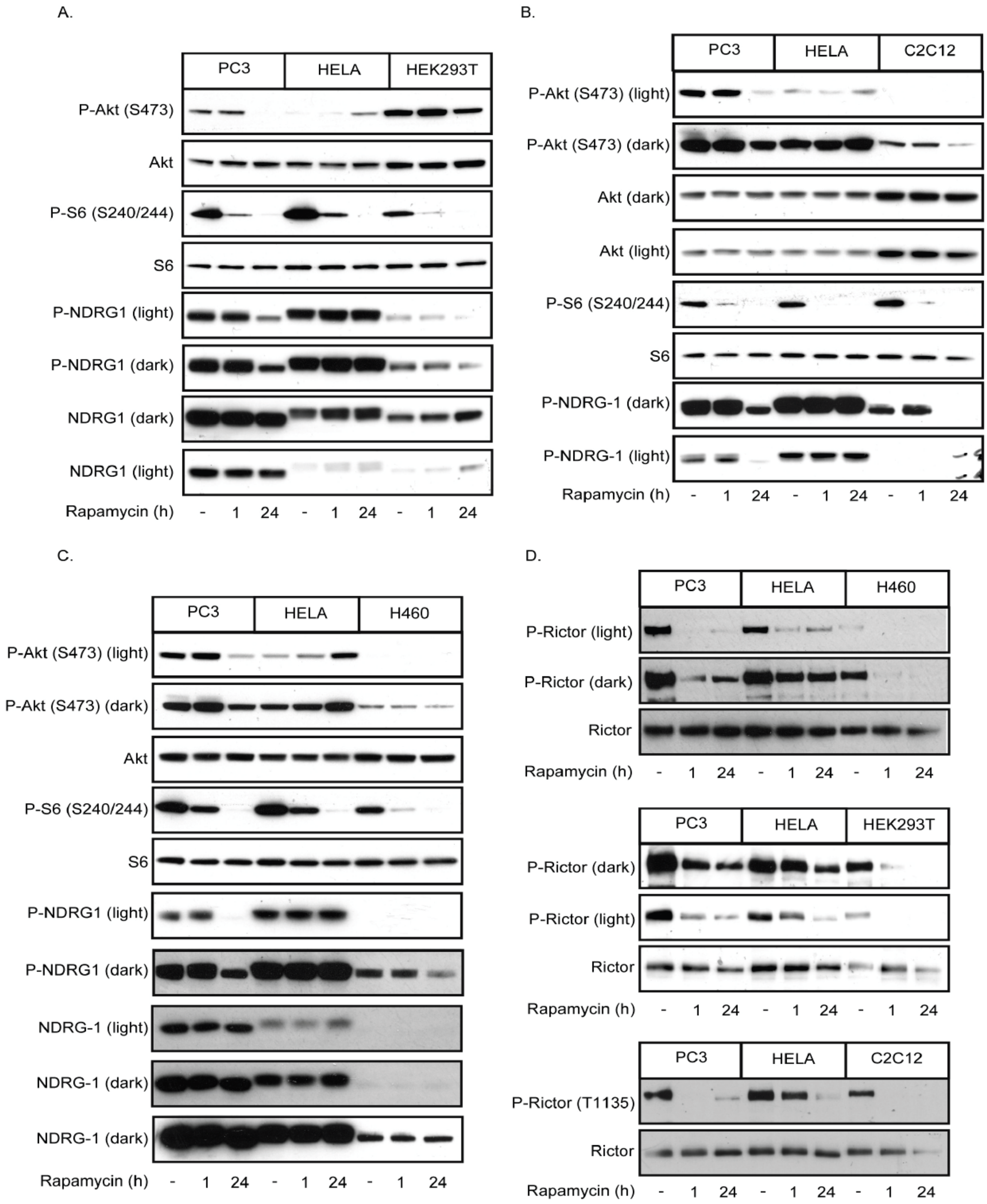

Figure S1. Rapamycin differentially inhibits mTORC2 in various cell lines. PC3, HeLa, HEK 293T, H460, or C2C12 cells were treated with 100 nM rapamycin for 1 or 24 h. Full western blots from Figure 1 are shown. The phosphorylation of specific mTORC1 substrates (P-S6K, P-S6) and mTORC2 substrates (P-Akt, P-NDRG1) were examined by western blot analysis (A, B, C). The phosphorylation of the mTORC1 substrate, Rictor, is inhibited by rapamycin in PC3, HeLa, HEK 293T, H460, and C2C12 cells (D).

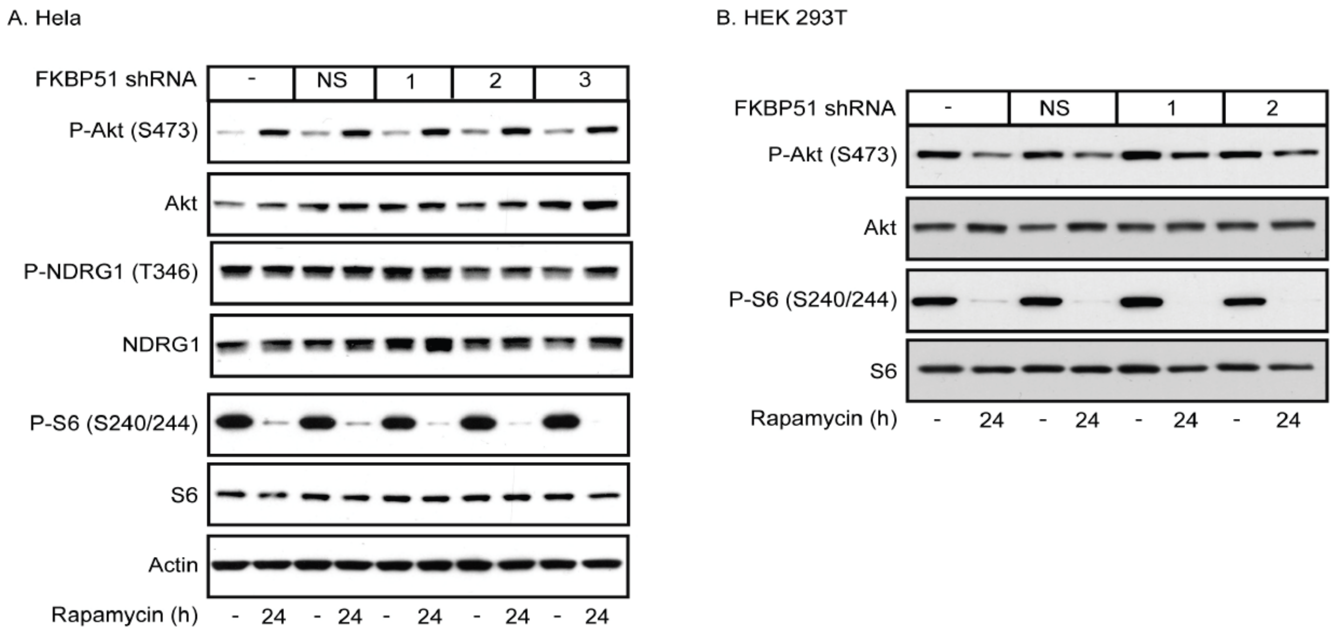

Figure S2. Knockdown of FKBP51 has no effect on mTORC2 inhibition by rapamycin in HeLa or HEK 293T cells. shRNA constructs directed towards FKBP51 were transfected into HeLa (A) or HEK 293T cells (B). Cells ex[ressomg shRNA 1 or 2 were treated with rapamycin for 24 h and the effects of knocking down FKBP 51 on mTORC1 signaling (P-S6) and mTORC2 signaling (P-Akt S473 and P-NDRG1) was examined by western blot analysis.

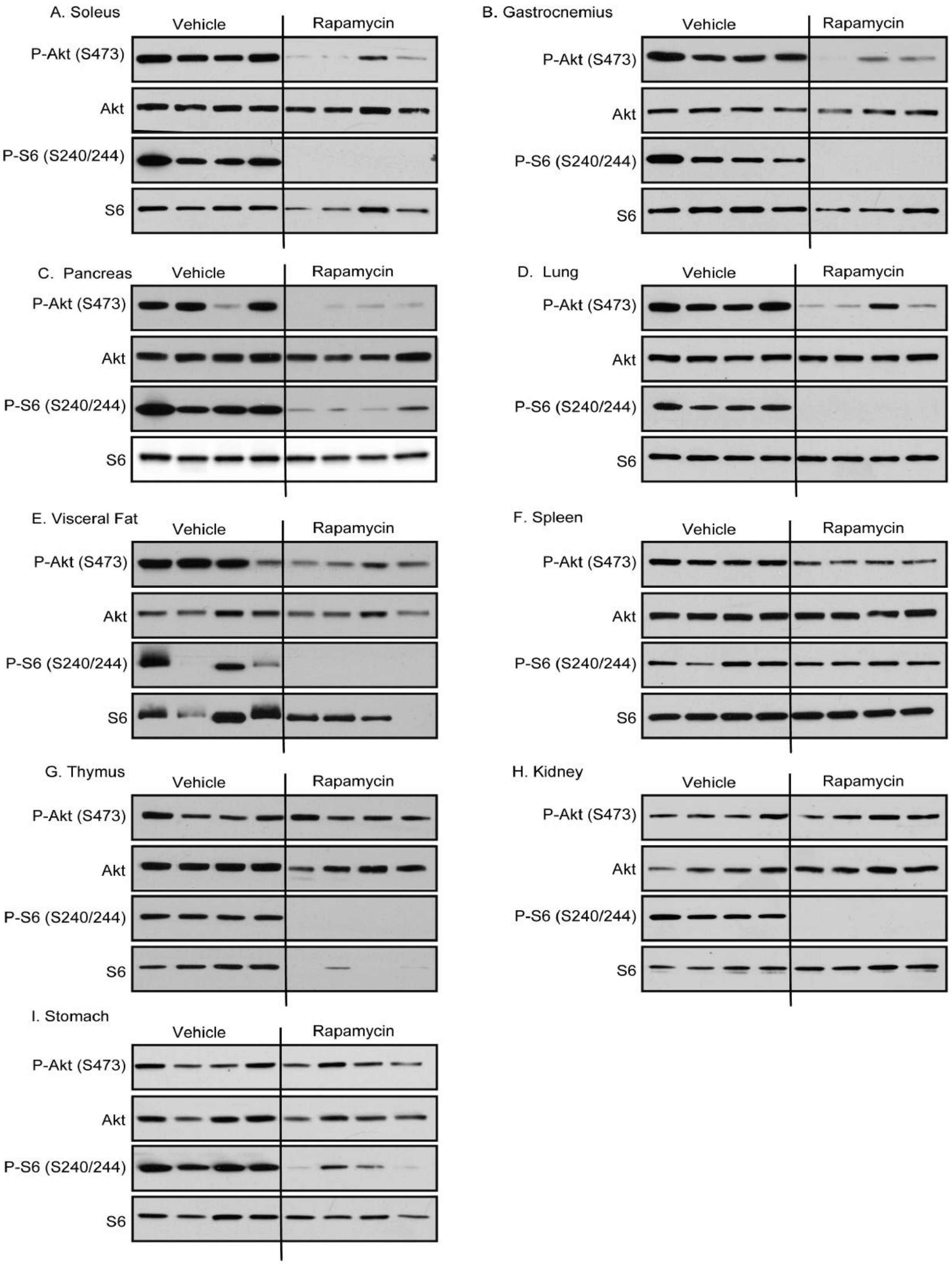

Figure S3. Inhibition of mTORC2 by rapamycin *in vivo* following an overnight fast and stimulation with insulin. Following rapamycin (8mg/kg) or vehicle treatment every other day for 3 weeks, mice were fasted overnight and stimulated with 15 min of insulin just prior to tissue harvest. The effects on mTORC1 inhibition (P-S6) and mTORC2 inhibition (P-Akt) were examined by western blot analysis.

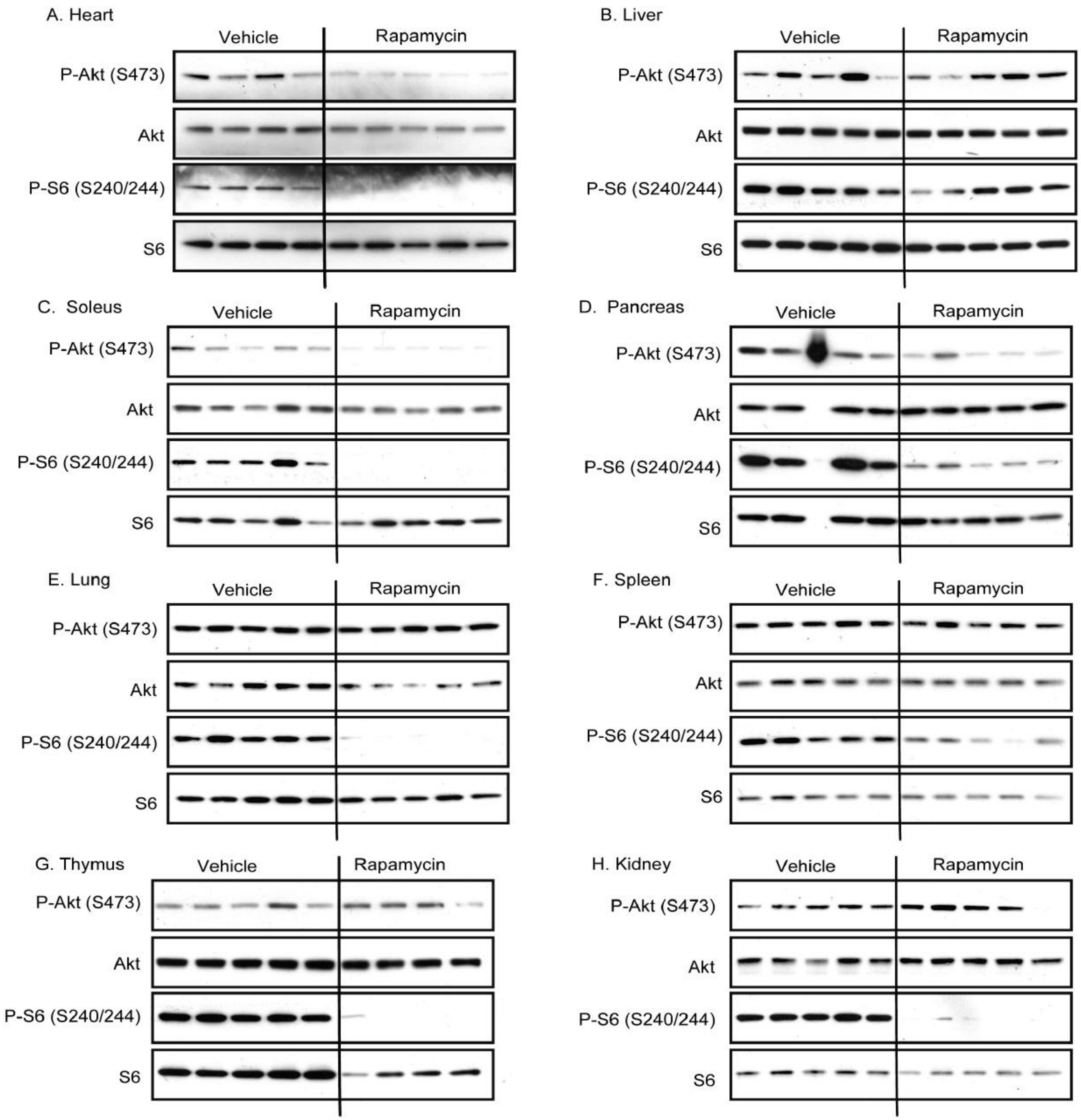

Figure S4. Inhibition of mTORC2 by rapamycin *in vivo* following a 6 h fast. Following rapamycin (8mg/kg) or vehicle treatment every other day for 3 weeks, mice were fasted for 6 hours and tissues were harvested. The effects on mTORC1 inhibition (P-S6) and mTORC2 inhibition (P-Akt) were examined by western blot analysis.
